# Unraveling Hematological Anomalies in DiGeorge Syndrome: A Retrospective Study of Thrombocytopenia and Mean Platelet Volume

**DOI:** 10.7759/cureus.78857

**Published:** 2025-02-11

**Authors:** Hamza Haj Mohamad, Abdelrahman K Nouh, Fatima AlAli, Sally AlNaeem, Fourat Aldchach, Hanan N Abouelkhel

**Affiliations:** 1 General Practice, University of Sharjah, Sharjah, ARE; 2 General Surgery, Al Kuwait Hospital, Sharjah, ARE; 3 Pediatric Genetics, Al Qassimi Women's and Children's Hospital, Sharjah, ARE; 4 Pediatric Hematology, Al Qassimi Women's and Children's Hospital, Sharjah, ARE; 5 Biostatistics, Aleppo University, Aleppo, SYR; 6 Pediatrics, Alexandria University, Alexandria, EGY

**Keywords:** bernard-soulier syndrome, digeorge syndrome, gpibb gene, mean platelet volume, tbx1 gene, thrombocytopenia

## Abstract

Background: DiGeorge syndrome, arising from chromosome 22q11.2 deletion including the *TBX1* gene, is known for its multifaceted developmental anomalies affecting the heart, immune system, and facial morphology. Despite extensive clinical characterization, hematological manifestations, particularly thrombocytopenia, remain underexplored. There is growing evidence of a potential association between DiGeorge syndrome and Bernard-Soulier syndrome, a rare platelet disorder characterized by defects in the GPIb-IX-V complex. This connection may be linked to the genetic location of the GPIBB gene, which encodes the GPIbβ subunit of the complex, on chromosome 22, where microdeletions are a hallmark of DiGeorge syndrome.

Objective: This study investigates the hematological profile of pediatric DiGeorge syndrome patients followed up in Al Qassimi Women's and Children's Hospital, Sharjah, focusing on platelet counts, mean platelet volume, and potential genetic links to GPIBB mutations on chromosome 22q11.2. The aim is to deepen understanding of thrombocytopenia in DiGeorge syndrome and its implications for clinical management.

Methods: A retrospective analysis of medical records identified eight pediatric DiGeorge syndrome patients diagnosed through fluorescent in situ hybridization (FISH) and microarray scanning, excluding cases with incomplete records or unrelated comorbidities. Uniform assessment of platelet parameters was conducted across all subjects.

Results: DiGeorge syndrome patients had a mean age of 5.1 years, with four males and four females. The mean number of complete blood counts (CBCs) per patient was 14.25 (range: 6-42). The mean platelet count was 194,295/µL and the mean mean platelet volume (MPV) was 10.8 fL. Thrombocytopenia and large platelets showed notable variability. Four patients had large platelets on 100% of their CBCs, while the lowest was 57%. Two patients had thrombocytopenia in >80% of CBCs, while the rest showed lower rates. One patient presented with immune bicytopenia that responded to immunosuppressive therapy.

Discussion: The findings underscore distinct hematological characteristics in DiGeorge syndrome. These insights into platelet abnormalities shed light on potential mechanisms underlying thrombocytopenia in DiGeorge syndrome. Specifically, the observed higher MPV and occasional presence of giant platelets suggest altered platelet production or function, possibly due to haploinsufficiency of gene within the 22q11.2 region, including GPIBB. The study's findings align with previous reports linking DiGeorge syndrome to thrombocytopenia and Bernard-Soulier syndrome features.

Conclusion: This study provides insights into hematological manifestations of DiGeorge syndrome, highlighting the role of GPIb-IX-V complex deficiencies. Further research is warranted to elucidate genetic interactions and optimize management strategies for thrombocytopenia in DiGeorge syndrome patients. Future investigations comparing proximal versus distal 22q11.2 deletions and their clinical implications could enhance our understanding.

## Introduction

DiGeorge syndrome, also known as 22q11.2 deletion syndrome, is a genetic disorder resulting from the deletion of a segment of chromosome 22. This deletion can lead to a range of developmental anomalies affecting multiple systems, including the heart, immune system, and facial morphology. T-box transcription factor 1 (TBX1) is a pivotal gene involved in embryonic development, particularly significant in DiGeorge syndrome. TBX1 plays a critical role in the normal formation of pharyngeal arch and pouch-derived structures during embryogenesis, and its deletion contributes significantly to the characteristic features of DiGeorge syndrome [1].

While clinical manifestations of DiGeorge syndrome, such as distinctive facial features, cardiac anomalies, thymic absence, cleft palate, and hypocalcemia are well-documented, further characterization of hematological manifestations remains underexplored. Thrombocytopenia has been frequently associated with DiGeorge syndrome in literature, often diagnosed as immune thrombocytopenic purpura (ITP) [2]. Recent studies suggest a potential link between DiGeorge syndrome and Bernard-Soulier syndrome (BSS).

BSS is a rare genetic disorder affecting blood clotting, marked by abnormally large platelets, thrombocytopenia, and extended bleeding duration. BSS is the result of genetic mutations encoding for GPIb-alpha (GPIbα), GPIB-beta (GPIbβ), and/or GPIX (GP9), which are three of the four subunits that make the GPIb-IX-V complex [3]. The GPIBA gene is located on chromosome 17, while the GPIBB gene is on chromosome 22, and GPIX is on chromosome 3 [4].

The GPIb-IX-V complex is expressed on the surface of platelets and is crucial for blood clot formation by facilitating platelet adhesion to the subendothelium, especially when the vascular subendothelium is exposed or a plaque ruptures. This complex plays a vital role in thrombosis by binding with von Willebrand factor (VWF) to initiate a signaling cascade that activates the platelet integrin GPIIb-IIIa, leading to platelet aggregation. The N-terminal of GPIbα is critical for platelet-mediated coagulation, providing binding sites for high molecular weight kininogen, factors XI, XII, and alpha-thrombin, also serving as a primary binding site for multiple ligands. It is also pivotal for the interaction between platelets and leukocytes in thrombosis and inflammation. Additionally, the GPIb-IX-V complex helps maintain platelet shape by linking the platelet surface to a network of actin filaments via the cytoplasmic tail of GPIbα. Mutations in the genes encoding this complex can lead to reduced platelet activation, defective adhesion, and impaired clot formation, explaining the presence of giant platelets in patients with BSS [5].

This study aimed to investigate the hematological manifestations in pediatric patients with DiGeorge syndrome, particularly focusing on platelet count and mean platelet volume (MPV). By analyzing clinical data and exploring possible underlying mechanisms, the study seeks to provide insights that could inform improved clinical management and treatment strategies.

## Materials and methods

We conducted a retrospective review of the electronic medical records of 10 pediatric patients diagnosed with DiGeorge syndrome, who were identified through genetic testing, including fluorescent in situ hybridization (FISH) and microarray scanning. The study was conducted at Al Qassimi Women's and Children's Hospital in 2024. Two patients were excluded from the study due to incomplete medical records or significant comorbidities unrelated to DiGeorge syndrome, resulting in a final cohort of eight patients.

Institutional review board approval was obtained from the Ministry of Health and Prevention Research Ethics Committee (approval MOHAP/DXB-REC/S.S.O /No. 156/ 2024) for the study prior to its commencement. Patient data were collected from the hospital’s electronic health records, which included a thorough review of medical histories, laboratory results, and clinical findings. Each patient had undergone multiple complete blood count (CBC) evaluations as part of their routine assessment. These CBC tests were used to assess platelet counts and MPV levels, which were specifically analyzed for features suggestive of BSS.

Platelet counts were measured using the Siemens Advia 2120i hematology analyzer (Siemens Healthineers, Erlangen, Germany), a standard method for determining platelet numbers in pediatric patients. Thrombocytopenia was defined as a platelet count below 150 x 10³/μL, and the presence of large platelets was indicated by an MPV greater than 11 fL [6]. In this study, MPV was used as a key marker for the assessment of platelet size, a potential indicator of BSS.

Data from the cohort were examined for evidence of hematological abnormalities, including thrombocytopenia, elevated MPV, and any potential correlations with DiGeorge syndrome and Bernard-Soulier syndrome. Statistical analyses were conducted to explore any significant relationships between DiGeorge syndrome features and BSS-associated hematological findings.

## Results

In Table 1, the data of the DiGeorge syndrome patients is presented. The average age of the patients at the time of data collection was 5.125 years, with an equal distribution of four males and four females. The number of CBCs per patient ranged from six to 42, with a mean of 14.25 CBCs.

**Table 1:** Demographic data of the DiGeorge patients with diagnostic method CBC: complete blood count, FISH: fluorescent in situ hybridization, aCGH: array comparative genomic hybridization

Patient Number	Age (years)	Gender	Number of CBC tests	Diagnostic method (probe number if FISH)
1	6	Male	14	FISH
2	4	Female	8	FISH (N25) 46,XX,ish del(22)(q11.2q11.2)(D22S75-)
3	11	Male	12	Array Scanning CytoScan: arr[GRCh38] 22q11.21(18,162,024_21,111,373)
4	3	Male	6	FISH
5	4	Male	7	FISH
6	3	Female	42	FISH (N25) 46,XX,ish del(22)(q11.2q11.2)(D22S75-)
7	4	Female	14	FISH
8	6	Female	11	Microarray-based aCGH Findings: arr[GRCh37] 22q11.21 (18729944_21589480)x1

**Figure 1:**
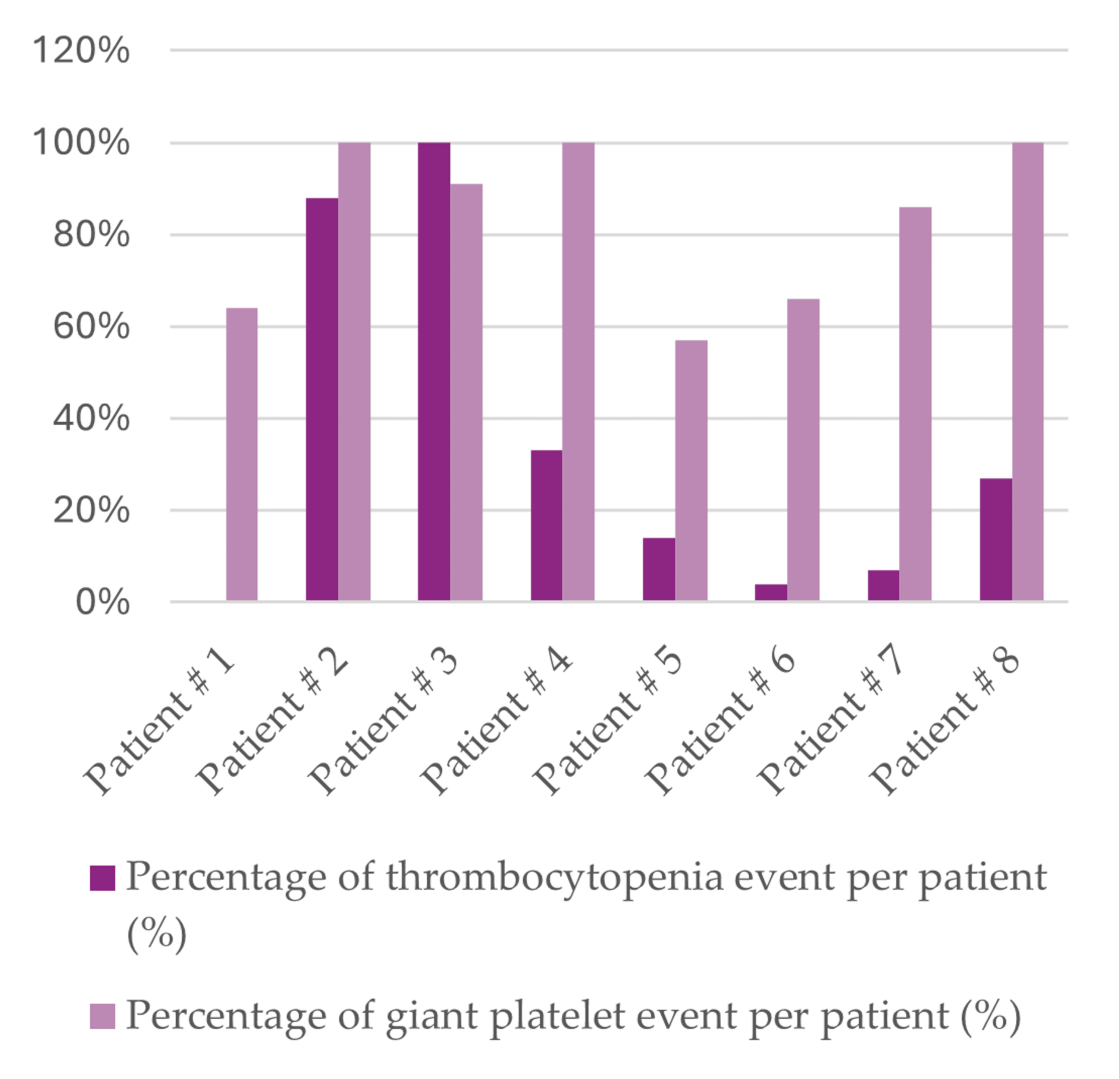
Comparing the percentages of thrombocytopenia and mean platelet volume (MPV) events per patient.

In Figure 1, the data for patients with DiGeorge syndrome reveals the relationship between this syndrome and thrombocytopenia events in addition to large platelet events. Seven out of eight patients had thrombocytopenia at least once during their follow-up period, while all of them had large platelets at least in 57% of their encounters. There was significant variability among patients. Notably, four patients (numbers 2, 3, 4, and 8) had large platelets in 100% of their CBCs (the percentage is calculated by dividing the number of CBC with large platelets over the total number of CBC for the patient). The occurrence of thrombocytopenia varied more widely among the patients. Only two patients (numbers 2 and 3) showed high percentages of thrombocytopenia, while the remaining patients exhibited lower rates.

Patients with DiGeorge syndrome had a mean platelet count of 194,295/µL (range, 4,000-490,000/µL) and mean MPV was 10.822 fL (range, 8.0-15.1 fL). Table 2 shows the mean and range of thrombocyte counts and MPV per patient. The estimated total platelet volume (thrombocrit) was not calculated in this study. Only one patient (patient number 3) had severe thrombocytopenia with a platelet count of 4, and a documented bleeding event (bleeding gums and epistaxis), associated with autoimmune bicytopenia. Blood smears showed giant platelets in one patient, while no giant platelets were observed in four patients, and the data was not available for three patients.

**Table 2:** Summary of key hematological parameters in DiGeorge syndrome patients. MPV: mean platelet volume

Patient Number	Percentage of thrombocytopenia event per patient (%)	Percentage of large platelets event per patient (%)	Blood Smear Showing Giant Platelets	Mean Platelet Count (/µL)	Platelet Count Range (/µL)	Mean MPV (fL)	MPV Range (fL)	Any Documented Bleeding Events (Yes/No)
1	0%	64%	N/A	293.29	198-566	9.34	8.10-10.70	No
2	88%	100%	No	136.5	109-151	12.43	11.70-13.00	No
3	100%	100%	Yes	49.67	4-147	11.87	8.30-15.10	Yes, Autoimmune bicytopenia
4	33%	100%	N/A	168.5	115-202	11.23	10.60-12.30	No
5	14%	57%	N/A	214.71	140-321	9.14	8.80-9.60	No
6	4%	66%	No	293.71	71-471	10.24	8.00-12.40	No
7	7%	86%	No	218.07	145-289	10.08	8.30-11.00	No
8	27%	100%	No	179.91	200-490	12.25	9.90-14.20	No

## Discussion

The current study aimed to investigate the hematological manifestations in pediatric patients with DiGeorge syndrome, focusing on platelet counts and MPV. The findings from this study highlight significant differences in platelet characteristics between patients with DiGeorge syndrome and normal population, with implications for understanding the underlying mechanisms and clinical management of thrombocytopenia in this patient population.

Our results are consistent with those of a previous study conducted in 2003 by Lawrence et al., which also reported lower mean platelet counts and larger MPV in patients with DiGeorge syndrome compared to the normal population, suggesting a common hematological manifestation of the chromosome 22q11.2 deletion [7]. Our study reported a slightly lower mean platelet count and higher MPV than those reported by Lawrence et al., however the ranges overlap widely.

The complexity of hematological manifestation in DiGeorge was underscored by Budarf et al., who reported on a six-year-old boy presenting with both BSS and features of DiGeorge syndrome [8]. The patient's clinical presentation included mild congestive heart failure due to ventricular septal defect (VSD), recurrent otitis media, easy bruising, and severe epistaxis. This patient exhibited thrombocytopenia with large platelets and a reduced platelet aggregation response, hallmark features of BSS. The FISH test confirmed the 22q11.2 deletion and platelet analysis via Western blot showed decreased GPIbα and absent GPIbβ proteins, marking the first reported case of BSS due to a mutation in the GPIBB gene in conjunction with a 22q11.2 deletion. This case suggests that patients with microdeletion syndromes (like DiGeorge syndrome) might be at risk for autosomal recessive disorders due to mutations in the non-deleted allele.

These findings suggest that haplo-insufficiency for genes within the 22q11.2 region, such as the GPIBB gene, might contribute to the observed platelet abnormalities. The GPIBB gene has been regionally assigned to 22q11.2 region using a somatic cell hybrid mapping panel [9] and by FISH [10]. In our study, four patients (numbers 2, 3, 4, and 8) were identified to have proximal deletions, and all of them exhibited 100% large platelets on CBC, suggesting features consistent with Bernard-Soulier syndrome. This is supported by the fact that the GPIBB gene is located within the proximal segment of 22q11.2, near the TBX1 gene. Patient number 3 array identified a heterozygous interstitial pathogenic variant loss of chromosome 22 at 22q11.21 of size 2,182kbps and encompassing 47 Online Mendelian Inheritance in Man (OMIM) genes, including TBX1(692054) and GP1BB(138720). This result confirms that this patient has defects in the gene responsible for BSS which may explain the bleeding episodes he had. Future research could focus on examining the specific deletion regions in DiGeorge syndrome patients, comparing proximal versus distal segment deletions. Since both the TBX1 and GPIBB genes are located in the proximal region, this raises the hypothesis that patients with proximal deletions may experience a higher incidence of bleeding events compared to those with distal deletions, potentially due to the deletion of the GPIBB gene.

The underlying mechanisms for thrombocytopenia and increased MPV in DiGeorge syndrome may involve several genetic factors within the deleted region. The GPIb-IX-V complex's role in platelet adhesion and clot formation is well-established, and haplo-insufficiency of the GPIBB gene could reduce platelet adhesion efficiency, leading to larger, less effective platelets. Additionally, other genes in the 22q11.2 region, such as TBX1, may theoretically contribute to the observed hematological abnormalities, but further research is required to clarify the exact genetic and molecular pathways involved. The potential association between thrombocytopenia, elevated MPV, and bleeding risk in DiGeorge syndrome is not well established. Although only one patient in our study experienced a bleeding event, the presence of increased MPV and occasional giant platelets suggests a possible, though unproven, predisposition to bleeding complications. This could be particularly important during surgeries or procedures requiring effective hemostasis. Ongoing monitoring of platelet counts and MPV may help identify patients at higher theoretical risk, but more research is needed to confirm these findings and guide clinical interventions.

On the other hand, thrombocytopenia in DiGeorge syndrome might be immune mediated. Patients with DiGeorge syndrome have small or absent thymus glands, with T-cell deficit and risk of recurrent infection, and immune dysregulation. The finding of other cytopenias (autoimmune hemolytic anemia/ autoimmune neutropenia) and/or other autoimmune diseases might suggest an immune etiology rather than the isolated megathrombocytopenia of Bernard Soulier-like feature. In addition, the high frequency of ITP in these patients could be the result of platelet membrane abnormalities as a consequence of quantitative abnormalities of GPIb [7].

In a big cohort of DiGeorge syndrome patients, Crowley et al. reported on higher risk for autoimmune diseases, mainly thyroiditis, juvenile rheumatoid arthritis (JRA) and ITP. ITP diagnosis was based on the findings of severe thrombocytopenia and response to immunomodulation [11]. In our study group, one patient (patient number 3) fitted in the diagnosis of immune cytopenia. He presented with severe thrombocytopenia, neutropenia, his bone marrow examination was hypercellular with no abnormal infiltrates, and he responded to intravenous immunoglobulin (IVIg) followed by a slowly tapering course of mycophenolate mofetil (MMF).

Similarly, several reports highlighted immune cytopenia in DiGeorge syndrome. Bruno et al. reported on immune pancytopenia in a child with DiGeorge syndrome who responded to treatment with steroids and reviewed another six cases with immune cytopenia [12]. Recurrent immune cytopenia was also reported by De Piero et al. in two patients who presented with autoimmune hemolytic anemia and thrombocytopenia [13]. In addition, Gu et al. reported on refractory cytopenia and autoimmune lymphoproliferative syndrome (ALPS)-like picture in a patient with partial DiGeorge syndrome who responded to sirolimus [14].

A key observation in our study group was that the only documented bleeding event occurred in the oldest patient, suggesting a possible link between age and bleeding risk in DiGeorge syndrome. However, this is a theoretical finding that requires further investigation. Future research should compare bleeding events in pediatric and adult patients to clarify this potential relationship.

This study highlights the need for further research into the hematological effects of the 22q11.2 deletion. Larger patient cohorts are required to validate these findings and explore gene interactions within the deleted region. Functional studies on platelet adhesion and aggregation in DiGeorge syndrome could offer deeper insight into these abnormalities [15]. A key limitation is the lack of bleeding time assessments, which are important in BSS. Including these measurements may improve our understanding of platelet function in DiGeorge syndrome and its overlap with BSS.

## Conclusions

In conclusion, this study highlights the complexity of hematological manifestations in pediatric patients with DiGeorge syndrome, focusing on platelet abnormalities such as thrombocytopenia and elevated MPV. Our findings confirm the significant prevalence of these abnormalities, consistent with prior studies, and suggest a potential link between the proximal 22q11.2 deletion and BSS. Patients with proximal deletions, including those identified in our cohort, exhibited a higher incidence of large platelets and may face an increased risk of bleeding events due to the deletion of the GPIBB gene, a critical component of the GPIb-IX-V complex.

This study underscores the importance of genetic and hematological evaluations in DiGeorge syndrome, particularly for patients with proximal deletions. These findings provide valuable insights into potential mechanisms underlying platelet dysfunction in this population and support the need for further research to explore the genetic and molecular pathways involved. Future investigations comparing proximal versus distal 22q11.2 deletions and their clinical implications could enhance our understanding and inform more tailored management strategies for these patients. Additionally, future research should examine the relationship between age and bleeding risk, as the only bleeding event in our study occurred in the oldest patient.
